# Assessing the feasibility of deep-seabed mining of polymetallic nodules in the Area of seabed and ocean floor beyond the limits of national jurisdiction, as a method of alleviating supply-side issues for cobalt to US markets

**DOI:** 10.1007/s13563-022-00348-w

**Published:** 2022-10-20

**Authors:** Alexander Cunningham

**Affiliations:** Centre of Energy, Petroleum and Mineral Law and Policy, University of Dundee, Dundee, Scotland

**Keywords:** Cobalt, Critical minerals, Deep-seabed mining, Polymetallic nodules, International Seabed Authority, L71, Y40, Q31, Q34

## Abstract

The growing importance of cobalt to the US economy has led to its categorisation as a critical mineral. Cobalt demand is increasing due to its requirement in lithium-ion batteries, which will significantly contribute to the energy transition. Supply is threatened for various reasons, primarily regarding supply chain concentrations, with the majority of the world’s cobalt originating in terrestrial deposits in the Democratic Republic of the Congo, and being refined in China. There remain environmental and ethical concerns over the present supply chain. Previous discussions around reducing cobalt’s criticality have suggested diversifying processing locations to reduce geographical and jurisdictional reliance where possible. This study assesses the viability of extracting cobalt from polymetallic nodules (PMNs) located on the deep-seabed in the Area, as an alternative strategy to reduce cobalt’s criticality. Assessments are made of the viability of PMN extraction considering ongoing barriers to introduction, contrasted with current arguments supporting PMN extraction. PMN mining offers a more stable and decentralised alternative to current cobalt supply. There exist impediments to its introduction, notably potential environmental impacts, which remain poorly understood. Technical and political restrictions must also be overcome. It is argued that the wider environmental benefits of increased cobalt supply from PMN mining may offset its detrimental environmental impacts. It is suggested that PMN mining be used in a wider strategy to improve supply security of cobalt to US markets.

## Introduction and global significance

Cobalt is a silvery-grey transition metal with diverse uses based on certain key properties, specifically ferromagnetism, relative hardness, wear-resistance when alloyed with other metals, low thermal and electrical conductivity, high melting points, and multiple valences (Slack et al. 2017). It is presently only extracted from terrestrial mining activities, with 131 cobalt-producing mines identified in 20 different countries as of 2020 (Brown et al. 2018; van den Brink et al. 2020). The vast majority of the global cobalt supply currently originates in the Democratic Republic of the Congo (DRC), which is responsible for 98,000 of the 142 tonnes (≈69%) produced in 2020 (Shedd 2022). Whilst there exist large cobalt resources on the deep-seabed in the form of polymetallic nodules (PMNs) (Hein et al. 2013), there are currently hard barriers to the immediate exploitation of these resources. Existing literature provides a solid foundation of the broader circumstances and challenges to the development of marine mineral resources (Kaluza et al. 2018; Toro et al. 2020b; Watzel et al. 2020), though there remains no specific literature pertaining exclusively to PMNs or marine-derived cobalt. This paper aims to explore the possibility for deep-seabed mining (DSM) of PMN deposits as a means of reducing cobalt’s supply risk to the US economy (thus its currently increasing criticality), from a political, economic, and legal perspective.

The global demand for cobalt is expected to increase exponentially in the coming decades, with high estimates anticipating a 10- to 20-fold increase by 2050 (Deetman et al. 2018), and even conservative expectations foreseeing a quadrupling of absolute demand over the same period (Tisserant and Pauliuk 2016). Worldwide primary production of cobalt was 123 kilotons (kt) in 2019, an overall decrease from the 144 kt in 2015 (Brown et al. 2021). Whilst production levels of cobalt appear to be relatively stable, its use in ever-more prevalent electric vehicles (EVs) and electronic equipment more generally is causing increases in the criticality of cobalt (Beatty et al. 2019). Optimistic projections of global cobalt supply forecast a minimum annual deficit of 24,000 kt by 2030, potentially rising to as high as 342 kt in more pessimistic scenarios (Alves Dias et al. 2018).

The electrification of currently fossil fuel-based energy sources is a major emerging trend worldwide (Mai et al. 2018). Resultantly, the storage requirement of lithium-ion batteries, of which cobalt is a key component, is causing increases in global demand for cobalt (Azevedo et al. 2018). This is primarily manifested in the drastic rise in demand for EVs, which comes as a response to mounting consumer concerns related to the environmental impact of greenhouse gas (GHG) emissions associated with fossil fuel usage of internal combustion engine vehicles (ICEVs) (Habib et al. 2018; Reitz et al. 2019). Roughly 25% of total GHG emissions in the EU are attributed to the transport sector, causing many to view EVs as a partial solution to curb the growing GHG emissions from ICEVs (Ajanovic and Haas 2018).

**Fig. 1:**
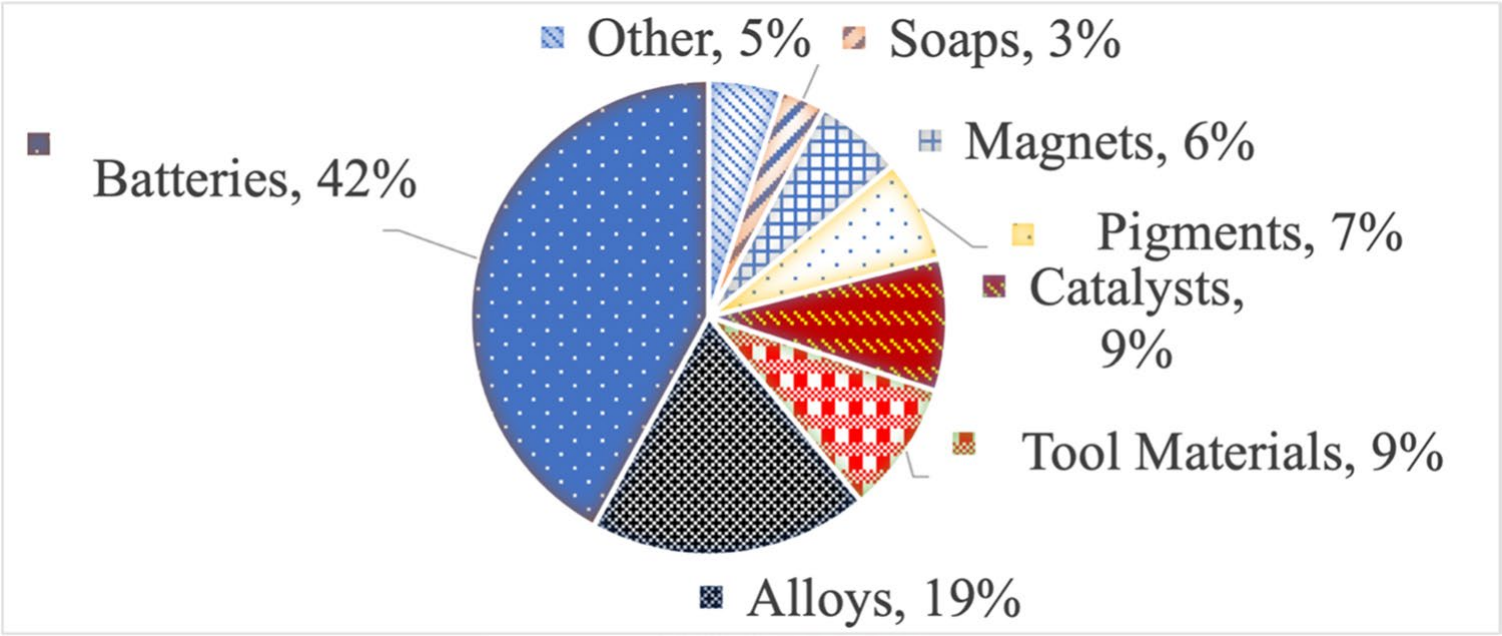
Global cobalt consumption, by end-use industry, 2017. After DeCarlo and Matthews (2019)

Cobalt is a vital component in many advanced economies, such as the USA, for its applications in modern technologies; the variety of which is detailed in Fig. 1. Cobalt’s unique properties as outlined above render its substitutability low, meaning that in many applications, further substitution other than that which currently exists would result in a deterioration of product performance and/or an increase in cost. Compounding cobalt’s criticality is its common extraction as a by-product of other metals, resulting in production levels being mainly driven by the market conditions of the principal metals such as nickel and cobalt, rather than the market’s requirements for cobalt (Slack et al. 2017). Consequently, it is considered a ‘critical’ mineral, defined by Executive Order 13,817 as (i) a non-fuel mineral or mineral material essential to the economic and national security of the USA, (ii) the supply chain of which is vulnerable to disruption, and (iii) that serves an essential function in the manufacturing of a product, the absence of which would have significant consequences for our economy or our national security (United States, Executive Office of the President [Donald Trump] 2017).

Whilst this paper has a greater focus on the supply of cobalt, it is accepted that demand has a significant influence on its overall criticality. Though cobalt is poorly substitutable in specific uses, emerging battery technologies containing no cobalt (Kotaich and Sloop 2009; Luo et al. 2022) have introduced debate over the importance of securing cobalt supply. Despite this, it is not believed that cobalt-free alternatives will eliminate demand for cobalt-bearing battery technology (Zeng et al. 2022), with expectations that demand for cobalt-free batteries in EVs will decrease relative to cobalt-bearing batteries over the coming decade (Azevedo et al. 2018).

## Criticality and Supply Alternatives

**Fig. 2:**
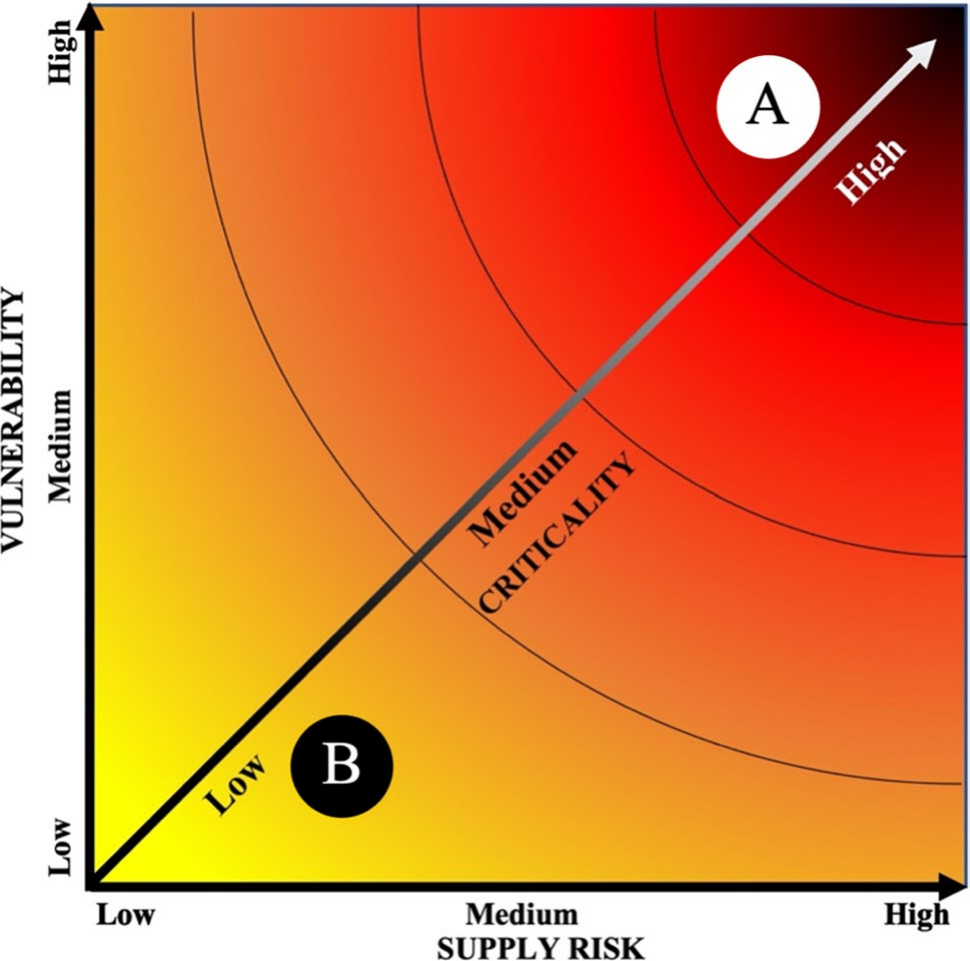
Criticality matrix. Degree of criticality increases away from origin. In this case, mineral A is more critical than mineral B due to its higher vulnerability (economic importance) and supply risk. After National Research Council (2008)

The concept of criticality in relation to minerals is inherently subjective (Kosmol et al. 2018); it is considered to be a function of levels of supply risk and the potential economic impact of supply restriction (National Research Council 2008). Degree of criticality can be assessed using the criticality matrix (Figs. 2 and 8), by which assessments are made for severity of supply risk (*x*-axis) and vulnerability (*y*-axis), with degree of criticality increasing away from the graph’s origin. Crucially, there is no objective value for criticality, with the matrix demonstrating three constitutive properties of criticality: (1) critical minerals are both essential for use and subject to potential supply threats. (2) Criticality is an amalgamation of values of various factors influencing supply risk and vulnerability. (3) Criticality is not pervasive, and is spatially, temporally, and contextually dependant (Buijs et al. 2012; Kosmol et al. 2018); the criticality of a mineral may differ greatly between different stakeholders, at different times, in different geographic, political, and economic environments.

### Polymetallic nodules

**Fig. 3:**
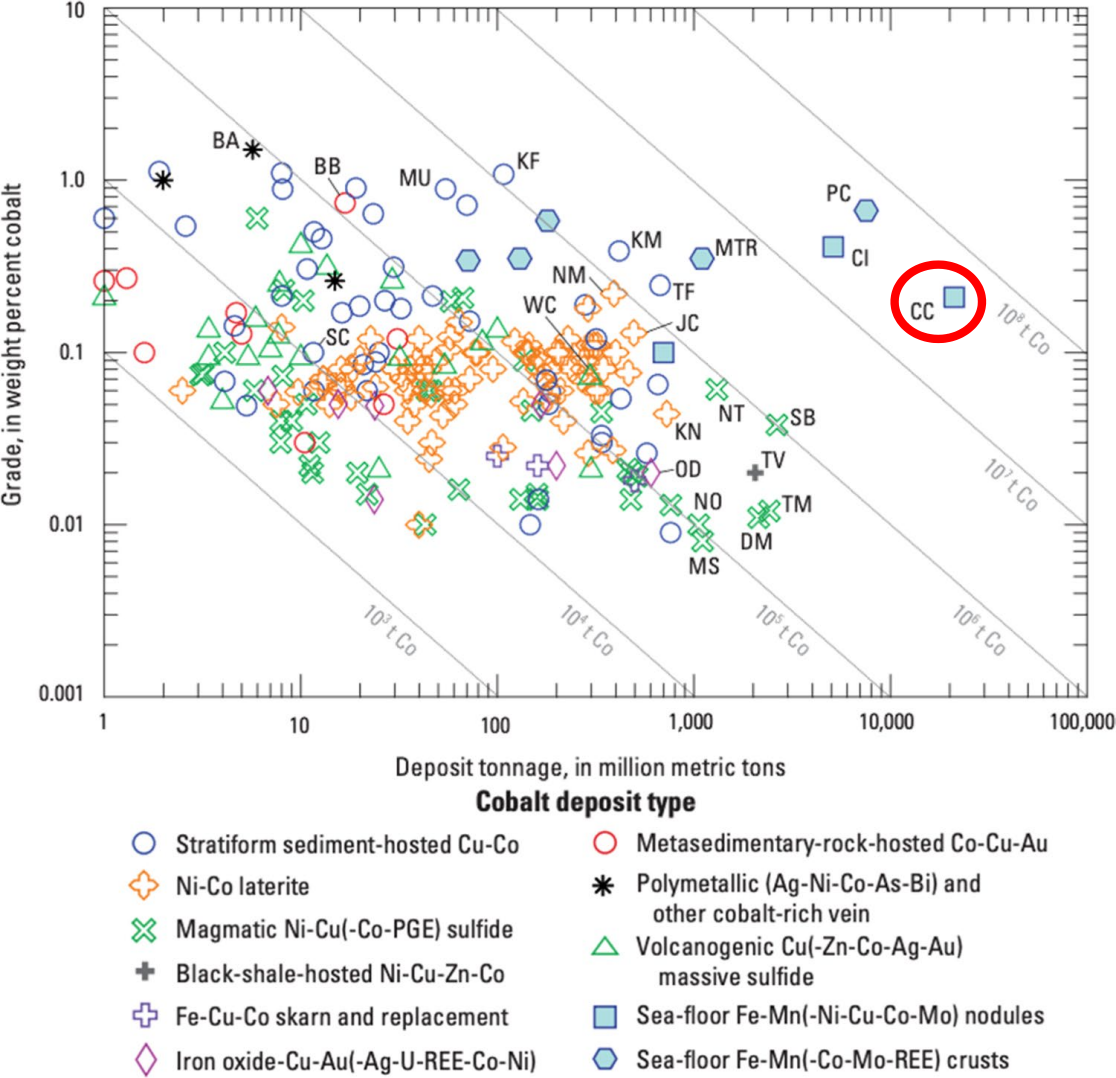
Grade-tonnage plot for 214 cobalt deposits worldwide. Grades and tonnages include production plus reserves plus other resources where known; reserve and resource data are from publicly available reports and company websites, but in some cases are not defined by a National Instrument 43–101 standard, Joint Ore Reserves Committee code, or similar mineral-resource classification scheme. Labelled deposits represent most of those containing more than 500,000 metric tons (t) of cobalt, many of the US deposits, some examples of the less common deposit types, and some that are discussed in the text. Small deposits—those with less than 1000 metric tons of cobalt—are not shown. Diagonal lines are isolines of contained cobalt, in metric tons. *BA* Bou Azzer (Morocco), *BB* Blackbird (Idaho), ***CC Clarion-Clipperton Zone (Pacific Ocean) (circled in red)***, *CI* Cook Islands Exclusive Economic Zone (Pacific Ocean), *DM* Dumont (Canada), *JC* Jacaré (Brazil), *KF* Kisanfu (Congo [Kinshasa]), *KM* Kamoto–KOV–Musonoi–Mupine deposits (Congo [Kinshasa]), *KN* Kalgoorlie Nickel (Australia), *MS* Mesaba (Minnesota), *MTR* Maderia-Tore Rise (Atlantic Ocean), *MU* Mutanda (Congo [Kinshasa]), *NM* Nkamouna (Cameroon), *NO* Northmet (Minnesota), *NT* Noril’sk Talnakh (Russia), *OD* Olympic Dam (Australia), *PC* Pacific prime crust zone (Pacific Ocean), *SB* Sudbury (Canada), *SC* Sheep Creek (Montana), *TF* Tenke Fungurume (Congo [Kinshasa]), *TM* Twin Metals (Minnesota), *TV* Talvivaara (Finland), *WC* Windy Craggy (Canada). Adapted from Slack et al. (2017)

Polymetallic nodules (PMNs) are potato-shaped metallic concentrations of primarily iron oxyhydroxides and manganese oxides that accrete around a nucleus, occurring as two-dimensional deposits on abyssal plains of all major oceans, and varying in size from less than one to tens of centimetres in diameter (Hein et al. 2020). Other than manganese, metals of economic interest contained within PMNs are nickel, copper, and crucially, cobalt. It is also noted that PMNs contain a variety of rare-earth elements (REEs) in promising quantities (Kuhn et al. 2017). The area with the largest known concentrations of the high-grade PMNs is the Clarion-Clipperton Zone (CCZ), situated in the abyssal South Pacific Ocean (Fig. 6), with the deposits considered to have potentially great commercial value (Beaudoin et al. 2014; Wedding et al. 2015). This area sits outside of the exclusive economic zone (EEZ) of any nation state and is therefore considered to lie within ‘the Area’ under the United Nations Convention on the Law of the Sea (UNCLOS), noted as the “seabed and ocean floor and subsoil thereof, beyond the limits of national jurisdiction [no further than 200 nautical miles from any coastal state]” (United Nations 1982). It is believed that the total contained cobalt within PMN deposits in the CCZ alone is 44,000 kt, more than three times greater than the global terrestrial reserve base of 13,000 kt, and over five times the global terrestrial reserves of 7500 kt^1^ (Hein et al. 2013). Figure 3 shows grades and tonnages for significant cobalt deposits worldwide.

### Methodology

This paper takes a pragmatic approach, and aims to explore the topic using a comparative methodology, assessing the potential for DSM of PMNs in contrast to current alternatives, specifically primary cobalt mined in DRC and secondary cobalt recovered as a by-product of other mining operations. Theoretic feasibility and promise of cobalt extraction from PMNs is examined by contrasting existing literature with precedents, trends, and research across economic, legal, and political fields. Consequently, quantitative analyses are only used where data is available for DSM and at least one alternative (primary or secondary recovery), allowing for comparison.

## Current barriers to deep-seabed mining of PMN deposits

For a variety of reasons, including fluctuating metal prices, recycling, and preferable terrestrial deposits, DSM of PMNs is yet to take place in a commercial setting (Sharma 2017). Though economically promising PMN discoveries were made in the 1960s and 1970s (Mero 1965; Price 1967; Bischoff 1976; Halfar and Fujita 2002), the implementation of UNCLOS (United Nations 1982) prohibited the claiming, acquisition, and exercise of rights with respect to minerals in the Area under Article 137. This Article confers the right to govern PMNs in the Area to the [International Seabed] Authority (ISA), which is yet to introduce regulations administrating exploitation, effectively prohibiting DSM of PMN deposits. The introduction of exploitation regulations, however, would unlikely result in commercial exploitation overnight; aside from the regulatory and political barriers to DSM, there are also economic (including technical) and environmental challenges which must be overcome before PMNs can be successfully extracted for commercial gain.

### Economic barriers to DSM of PMNs

Though some may see the motivation for mining operations as to obtain supplies of minerals for their home countries’ consumers or for their own integrated supply chains (Humphreys 2013), the main objective, or bottom line, of private mining companies is to earn profits for their shareholders (Crowson 1986). Crowson argues that every producer aims to have costs in the industry’s lowest quartile, in order to remain internationally competitive against their peers and ultimately ensure the maximisation of value for their shareholders. Given that exploration companies only have a finite quantity of resources available for investment, they must choose not only a project that is profitable, but one that they perceive to be *more* profitable than alternatives, should they choose to invest at all. Alternatively, in the interest of a state’s wider economy, governments such as that of the USA may consider subsidising or even wholly operating PMN mining activities, with the intention of securing the supply of a critical mineral such as cobalt (Humphreys 2014). This, however, cannot be seen as a solution to economic barriers to DSM. Rather, it should be viewed as a government artificially reducing deterrently high costs. Ultimately, the industry cannot be seen as truly economically viable unless PMNs can be routinely extracted by private enterprises for profit, without relying on the support of financial government intervention.

Repeated evaluations on the economic potential of DSM for PMN deposits have cemented its status as a serious avenue of exploration (Sharma 2017, and references therein). It is acknowledged that whilst it can be seen as an ‘alternative’ to terrestrial mining, the costs of a company with solely terrestrial mining experience adapting to operating conditions as different as those in abyssopelagic environments are likely to be greater than the marginal gains in comparison to developing terrestrial deposits (Rademaekers et al. 2015).

The economics of a given activity are assessed using cost–benefit analyses, comparing the required inputs against the potential outcomes from an economic perspective to determine financial feasibility. Though any analysis should aim to provide a financially quantifiable answer, many of the inputs and outcomes involved in DSM analyses are intangible, giving potentially large uncertainties (Volkmann and Osterholt 2017; Roth et al. 2018a). This also raises the question of negative externalities — economic, social, and/or environmental costs generated by operators which are borne by others. Assuming that the production of negative externalities is not financially punished by regulators, they rarely manifest as immediate financial consequences and are thus often not considered to be negative costs on companies’ balance sheets (Unerman et al. 2018). In the short term, negative externalities may affect an operator’s *willingness* to engage in DSM activities, though they are not considered to affect its *ability*. These externalised costs, by definition, are unlikely to immediately affect an operator’s bottom line, and therefore are not considered to be an economic barrier to DSM of PMN deposits. As this paper is not intended to provide intricate econometric evaluation, the exact size of the economic obstacle to DSM is not scrutinised. Rather, each economic barrier is outlined as a distinct challenge for operators to overcome.

The inputs to a mining company concern the abstract logistics of locating and then transferring metals captured in PMNs to either terminal markets or integrated supply chains. The deposits must first be found and evaluated, using prospecting and exploration techniques, at an estimated per concession cost of US$10 mn per year for approximately 5 years (Cardno 2016). The prospecting and exploration costs associated with PMNs derive from performing the detailed bathymetric surveys to determine suitability of the seafloor topography for mining, as well as nodule coverage. Sampling must then take place to determine metal grades and abundance, which allows for the market value of the deposit to be calculated (Cardno 2016). This calculation can include a wide margin of error, especially where a project relies on multiple metals for revenue, and even more so if prices of said metals are highly volatile, or there exists the prospect of wide-scale end-use substitution, i.e. cobalt, in the case of cobalt-free batteries. There is currently adequate technology to complete this stage (CORDIS 2020; DEME 2021; Lipton et al. 2021), reducing the need for costly development of new technologies.

Though this paper is primarily legal and economic in focus, it is acknowledged that the technical aspect of mining is one of the pivotal aspects of its economic success. Until legally permissible under ISA regulations, which are yet to be published (the “Regulatory barriers to DSM of PMNs” section), the technology required to harvest and lift the nodules cannot be proven in a commercial setting. Though partial adaptation of technology used in space (Jasiobedzki and Jakola 2008) and subaqueous hydrocarbon extraction (Atmanand and Kathiroli 2008) may provide a nucleus from which PMN extraction technology can be developed, there are inherent differences in PMN deposits which make DSM more costly (Cardno 2016). With the suitability of repurposed equipment for PMN mining not yet known, certain technical challenges remain unsolved; whilst specific components may be capable of individually executing tasks to a sufficient standard as initially intended, it is not yet known what will work, to what extent, and what will be the consequences on the wider system of shortcomings of a different component.

Designing and building equipment can be expensive and time consuming, especially when each aspect of an entirely novel operation requires some degree of new technology. This begins at support vessels, which require the capability to manage the unprecedented activity from the surface, acting as the interface between the subaqueous operations and transport ships. Though this will require specialist equipment, a precedent is provided in the Solwara 1 Production Support Vessel (Schuler 2018). Arguably the most important technology is the excavation tool itself, which is expected to resemble a combine harvester (Heffernan 2019). This technology is currently in development by specialist marine engineers, and will likely feature many of the same aspects of the DSM vehicles used in the Solwara 1 project.^2^

Further technological requirements are the risers and lifting systems, which transport the minerals as far as 6 km from the seafloor to the surface (Yamazaki 2017). These systems may require the most specific design due to the unique nature of the deposit, and have been the most costly to develop in other related projects (Cardno 2016). Finally, the material must be prepared for transport, which requires dewatering and cargo handling systems. As with other aspects of DSM technology for PMNs, lack of precedent requires the need for purpose-built technology specific to the deposit, due to the unique challenges brought about by the nodules’ form (Lipman and Yu 2019).

**Fig. 4:**
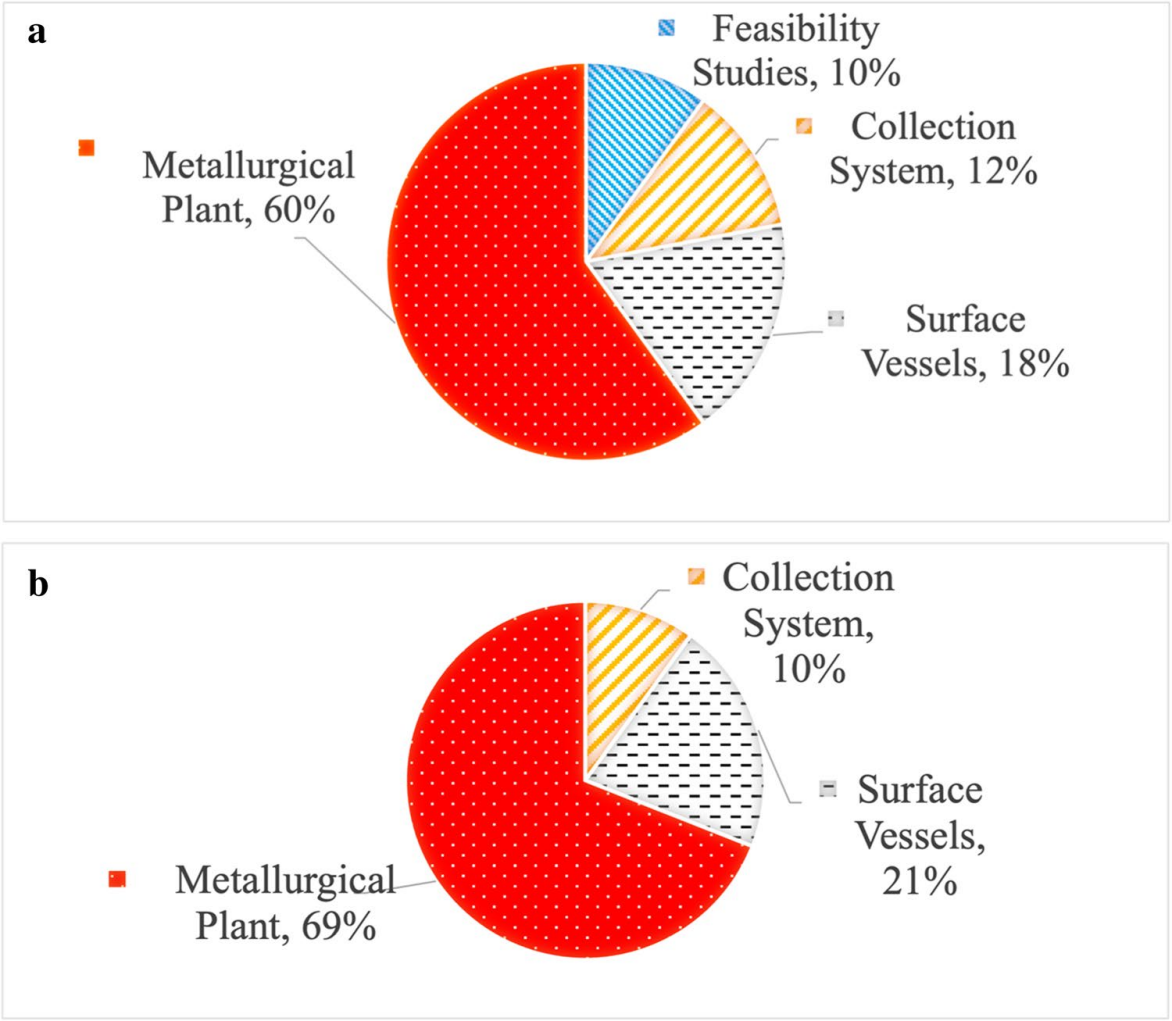
**a** Breakdown of total expected capital expenditures (CAPEX) for a DSM operation to exploit a PMN deposit. After Roth et al. (2018a). **b** Breakdown of annual expected operational expenditure (OPEX) for a DSM operation to exploit a PMN deposit. After Roth et al. (2018a)

Costs at this stage are all considered to be up-front, which must be completed prior to extraction. Feasibility studies are to be undertaken in the preliminary stages, followed by the procurement of specifically developed collection systems, transport ships, and a bespoke onshore metallurgical plant. Roth et al. (2018a) expect the capital expenditure (CAPEX) of a project to total between US$3–4bn, with most of this coming from the metallurgical plant^3^ (Fig. 4a). Volkmann and Osterholt (2017), however, believe the likely CAPEX to be around US$1.2–1.5bn. The ranges of and variations between these two figures demonstrate the vast economic uncertainty surrounding DSM projects.

Following initial CAPEX comes operational expenditure (OPEX), which denotes annual running costs of the project (Fig. 4b). Again, the majority of this expenditure is allocated to the metallurgical plant, an economic challenge in and of itself. There are at least 20 different metallurgical processes available to extract metals from PMNs based on five primary methods. Each has its own benefits and drawbacks, with varying levels of recovery of each target metal (Das and Anand 2017). This means that, assuming that the operator is responsible for the whole project from mine to market, they must decide which process to use. This involves determining whether to extract manganese alongside copper, nickel, and cobalt^7^, as well as detailed market forecasts for each metal, to ascertain the likelihood of continued economic viability. The price of a target metal may decrease drastically, making it subeconomic, whilst it may transpire that the price of a previously uneconomic constituent metal, i.e. titanium, increases exponentially, making it theoretically economically viable to extract. The sunk costs involved with the metallurgical plant render later changes to extraction method prohibitively expensive, requiring the operator to make a lasting decision based on market expectations, long before any revenue is generated.

**Fig. 5:**
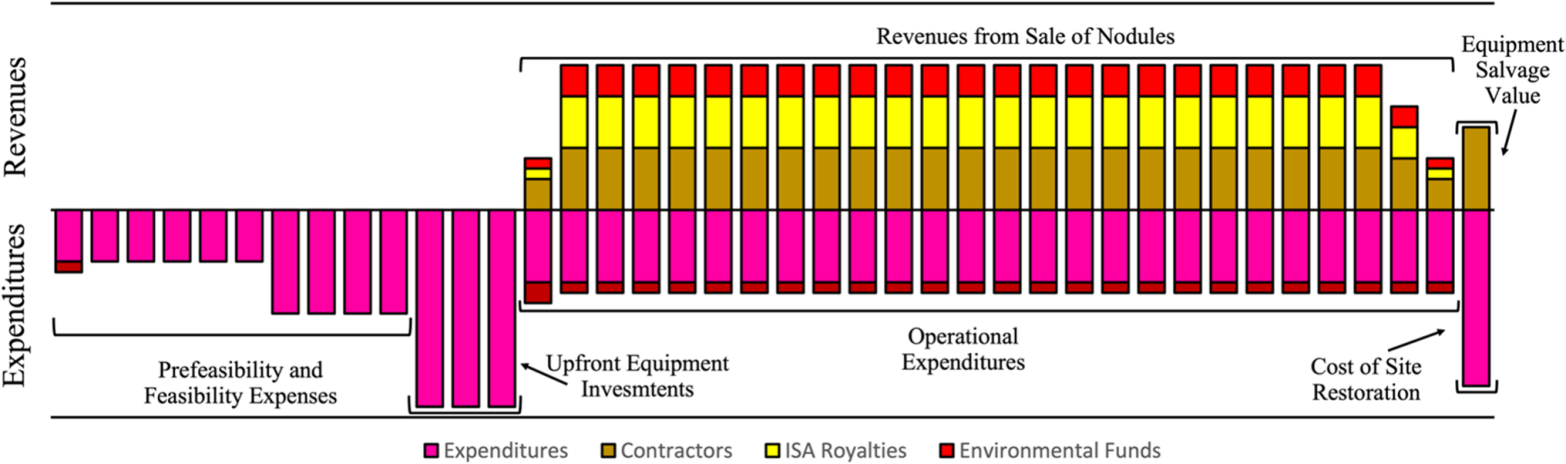
Projected expenditures and revenues profile for the lifetime of a hypothetical DSM operation to exploit a PMN deposit. Each bar represents a yearly total expenditure/revenue. Vertically not to scale. After Roth et al. (2018b)

Figure 5 shows a projected lifetime cashflow profile for a DSM operation. Though this does not differ from a typical cashflow profile for a mining project (Banda 2019), it must be noted that DSM operations have never been undertaken on PMN deposits at scale, and thus are considered a much higher risk investment than terrestrial mining projects. This may deter investors, with banks potentially unwilling to provide loans (Roth et al. 2018b). Furthermore, failed DSM projects such as Solwara 1 may validate concerns over the viability of DSM for PMNs^2^.

It is believed that PMN mining projects can be profitable under favourable conditions, with potential project earnings before interest, tax, depreciation, and amortisation of US$3.735bn (Abramowski et al. 2021). Despite this, profitability is far from guaranteed (Koschinsky et al. 2018; Volkmann et al. 2018), underscoring the financial risks associated with PMN mining. Compared with terrestrial mining projects, DSM is considered to be potentially higher return although it undoubtedly brings higher risk to investors (Roth et al. 2018b). This is primarily due to its unproven status, as well as various economic barriers such as the requirement for purpose-built technological equipment and metallurgical plants, multiple possible extraction paths (each with unique economic benefits and drawbacks), the need to rely on the economic health of multiple metals for profitability, and uncertainty over mandatory deductions such as taxes and environmental funds (Volkmann et al. 2018).

### Environmental barriers to DSM of PMNs

Possibly the greatest restriction to commercial DSM activities is the environmental question: can the environmental impacts be justified by the overall benefits? This aspect surrounds environmental impacts of DSM of PMN deposits, rather than the impact of the environment on mining operations, which is discussed in the “Economic barriers to DSM of PMNs” section. In order to solve this conundrum, the environmental impacts must first be known. This section considers environmental barriers to PMN mining by assessing the specific environmental impacts of PMN mining where possible, though acknowledging similarities between PMN mining and other DSM activities.

The deep sea (> 200-m depth) represents 95% of the global biosphere by habitable volume, though an overwhelming proportion of it remains totally unexplored (Miller et al. 2018, and references therein). As such, it is accepted that there are vast knowledge gaps regarding deep-sea ecosystems and their responses to anthropological activities (Ramirez-Llodra et al. 2011). Even more poorly understood, by virtue of this initial knowledge gap, is the impact of DSM on deep-sea ecosystems, resulting in strong opposition to DSM activities.

It is expected that DSM will likely affect marine ecosystems at every depth at which it operates, including the seabed, the water column, and the surface (Kaikkonen et al. 2021), as well as producing terrestrial environmental disturbances as a result of the processing of the PMNs themselves (Habashi 2012). This being said, some early predictions have suggested that whilst the environmental consequences will be far reaching, impact will decrease away from the mining site (Boschen et al. 2013; Van Dover 2014). In spite of this, the annual mining footprint will likely be within the region of hundreds to thousands of km^2^ per year (Oebius et al. 2001; Jones et al. 2017), and it is believed that disturbance from a single mining operation could be 2–fourfold greater than its initial footprint (Smith et al. 2020).

A fundamental issue with DSM activities is the much lower current velocity in deep-sea environments to other aquatic environments (Morgunov et al. 2014; Shanmugam 2021), which results in lower rates of sedimentation and nutrient replenishment (Hjulström 1935; Chen 2008). This, in turn, amplifies any physical effect that DSM has on the environment in terms of both time and intensity, as a result of artificial turbidity and sediment plume generation. Physical evidence of experimental PMN collection activity, in the form of equipment tracks in seafloor sand, was still present 37 years after the activity had taken place, demonstrating the environment’s poor recovery rate (Khripounoff et al. 2006). When it is taken into account that epifaunal densities of PMNs are over twice that of PMN-free areas, and that PMN mining is likely to remove all epifauna as well as have significant impacts on benthic fauna in the water column (Vanreusel et al. 2016), the immediate and severe effect of DSM on the local environment becomes difficult to justify (Jones et al. 2017).

Though some suspected environmental impacts have been well substantiated, it is still believed that the impacts are grossly miscalculated. This is due to underestimating DSM footprints relative to habitats targeted, as well as large knowledge gaps regarding the sensitivity, biodiversity, and dynamics of deep-sea ecosystems (Smith et al. 2020). Critically, there are still many unknown unknowns, meaning that the initial baseline assumptions against which to compare are far from sufficiently comprehensive to draw even remotely accurate conclusions regarding the environmental impacts of DSM activities (Heffernan 2019).

These huge data gaps represent a significant challenge for the DSM industry, with the majority of opposition resulting from environmental concerns (Miller et al. 2018). The ISA’s permitting procedure works using a precautionary approach, requiring that prospecting and exploration activities should not be undertaken “if substantial evidence indicates the risk of serious harm to the marine environment” (International Seabed Authority 2013), though this may be seen as a principle of ‘harmless unless proven harmful’. Exploitation permits, according to the Draft Regulations, may be granted if the environmental plans “provide for the effective protection of the Marine Environment in accordance with Article 145 of the Convention [UNCLOS], including through the application of a precautionary approach and Good Industry Practice” (International Seabed Authority 2021). Article 145 requires that “necessary measures be taken…to ensure effective protection for the environment from harmful effects which may arise from such activities” (United Nations 1982). Given that there is insufficient baseline information available, the ISA may, in theory, justifiably grant PMN prospecting, exploration, and/or exploitation permits for environmentally harmful activities, worrying many opponents of DSM practices.

Alternatively, it could be argued that the ISA does not have the same economic incentives to grant permits for potentially environmentally detrimental activities in the way a national government may (Waye et al. 2010). This argument is strengthened by the ISA’s mandate “to ensure the effective protection of the marine environment from harmful effects that may arise from deep-seabed related activities” (International Seabed Authority 2021), which may reduce the ISA’s scope for interpretation of environmental protection, encouraging more stringent regulatory controls in regard to permitting. Some academic opinions refute this, believing that the ISA has more of an economic focus than is openly suggested. It is argued that “protection of the marine environment was not the driving force behind the development of the international seabed mining regime. Rather, the focus was on establishing a regime to regulate access to seabed minerals and the sharing of benefits from their exploitation” (Jaeckel and Rayfuse 2017). This argument is formulated on the premise that the ISA are in the process of developing a permitting framework for an activity of which the environmental impacts are far too uncertain to develop regulations ensuring the protection of the environment. As a result of this, there is currently a lack of transparency in the decision-making process regarding exploitation permits. The Draft Regulations require an Environmental Impact Statement, an Environmental Management and Monitoring Plan, and a Closure Plan to be included in the final permit application (ISA 2018). These documents are scrutinised by the ISA’s Legal and Technical Commission, who must consider comments from members of the Authority as well as Stakeholders, though unanimous consent is not required. The opacity regarding exact criteria for acceptance, as well as a lack of baseline environmental information in which to ground the environmental submissions, are a notable barrier to DSM activities in the near future.

Further to these environmental barriers is the additional unanswered question of liability in case of damage. With many potential environmental impacts yet to be discovered, there is a distinct possibility that DSM of PMN deposits will create some degree of environmental degradation which will remain undetected until after the damage is done. There is, as yet, no clearly defined landscape for liability in the event of environmental damage (MacMaster 2019). As pollutants can travel much further in water, the extent of the damage may be significantly higher than that of a similar terrestrial incident (Likens and Bormann 1974; Halfar and Fujita 2002). This, in turn, increases the potential severity of any environmental damage arising from DSM activities, and thus the cost of indemnity insurance for operators, sponsor States, or flag States^4^ — it has even been suggested that the ISA may bear some liability for environmental impacts related to DSM (MacMaster 2019). This vast legal uncertainty must be resolved prior to commercial DSM activities beginning, or else operators, sponsor States, and flag States risk having to ultimately bear the cost arising from the production of any negative externalities related to DSM activities in which they are involved.

### Regulatory barriers to DSM of PMNs

As noted in the “Polymetallic nodules” section, mineral resources within the Area, which includes PMN deposits, are under the jurisdiction of the ISA, according to UNCLOS (United Nations 1982). Of 193 UN Member States, 167 have ratified the Convention, though notably, the USA is not one of them (United Nations 2021), as discussed in the “United States specific barriers” section. Prospecting and exploration aspects of DSM are currently governed by the Regulations for Prospecting and Exploration for Polymetallic Nodules in the Area (International Seabed Authority 2013), under which 18 contractors have been granted licences for PMN exploration so far (International Seabed Authority 2021). Despite this, though the ISA’s Draft Regulations on Exploitation of Mineral Resources in the Area (ISA 2018) have been developed by their Legal and Technical Commission, they are yet to be finalised and introduced (Shabahat 2021). It was expected that the Exploitation Regulations would have been completed and introduced in 2020, though the COVID-19 pandemic delayed this (Kuykendall 2021), with no introduction as of August 2022.

In reaction to growing frustration with ongoing delays and alleged pressure from investors, the government of Nauru, an ISA Member, triggered a clause in UNCLOS mandating the introduction of exploitation regulations within 2 years (Morse 2021). Section 1, Paragraph 15 (b) of the Agreement relating to the implementation of Part XI of UNCLOS states that “If a request is made by a State … the [ISA] Council shall … complete the adoption of such rules, regulations and procedures within two years of the request” (United Nations 1994). Consequently, the ISA must introduce the regulations necessary for commercial DSM activities by 9 July 2023. If the Regulations are not published by the deadline and an application for an exploitation contract is pending, the ISA Council must “consider and provisionally approve it on the basis of provisions and norms of UNCLOS” (Willaert 2021b).

The regulatory barrier in this instance is, again, based in the uncertainty of the prospect of a multitude of potential outcomes, with no clear indication as to which will prevail. There are growing calls from various ISA Member States for a Moratorium on DSM, with the launch of the Alliance of Countries for a Deep-Sea Mining Moratorium taking place at the UN Ocean Conference in Lisbon in June 2022 (United Nations 2022). In the event that such a moratorium is not imposed on DSM and the current trajectory is maintained, there is no guarantee that the final Regulations will resemble the Draft Regulations, leaving the prospect that DSM activities could end up being undertaken without the necessary environmental provisions, for example. Though this acceleration of the introduction of the Regulations is seen as a positive by many contractors, there is concern that it may result in more stringent regulations than if the ISA had produced them without this new time constraint (DSM Observer 2020b).

In addition to this regulatory uncertainty, operators understand that the motivation for DSM projects comes from the revenue gained from the sale of recovered metals. They must therefore be aware that the demand for metals contained in PMNs, especially cobalt, is being driven by growing environmental conscience from consumers (Alves Dias et al. 2018; Chen et al. 2019). If the Regulations are deemed to be insufficiently protective against potential environmental damage associated with DSM, purchasers may be deterred by perceived association with a practice considered by customers to be environmentally damaging. BMW Group, Volvo, Samsung, Google, and Philips all recently signed a letter calling for a moratorium on DSM (World Wildlife Fund 2021). The statement committed the signatories to abstain from procuring any metals from DSM-related sources, due to concerns that the environmental risks are too poorly understood for exploitation to safely take place (the “Environmental barriers to DSM of PMNs” section).

The structure and organisation of the ISA cannot be ignored when considering potential developments, or hindrances thereto, towards exploitation of PMNs. Part XI, Sect. 4, Subsection A of UNCLOS (General Provisions of the Authority), provides the foundation for the structure of the ISA, including the composition of its 3 principal organs (Assembly, Council, Secretariat). Whilst the Assembly is considered the supreme organ of the Authority, given its composition of all States Parties as members, the Council consists of 36 members^5^ elected by the Assembly. Despite the appearance of fair democracy, the Assembly may only elect Council members from specific categories, skewing the composition of the Council towards larger economies and those with potentially conflicting interests in DSM. Council members, therefore, may be more likely to vote in self-interest, rather than that of all mankind, as discussed in the “Political barriers to DSM of PMNs” section. The likely disharmony of stances could frustrate progress in the decision-making process on the introduction of regulations, as well as the allocation of exploration/exploitation permits, should such a decision be deemed to conflict with individual Member States’ interests.

#### United States specific barriers

Despite being an incredibly broad treaty governing all aspects of maritime activities, the primary objection to ratification of the UNCLOS from the USA was related to its effective prohibition of DSM (Bateman 2007). The USA has “repeatedly demonstrated its intent to be bound by the provisions of UNCLOS not relating to Part XI, which prohibits mining on the deep-sea beds” (Larkin 2010), with the belief that it “cannot effectively protect its interests within multilateral organisations” (Antrim 2015). States Parties to UNCLOS are *ipso facto* members of the ISA, meaning ISA rulings and regulations apply to all UNCLOS signatories (International Seabed Authority 2022). Consequently, US firms are either able to claim and exploit PMNs in the area without ISA approval, or are legally unable to exploit any PMNs beyond national jurisdiction, depending on legal interpretation (Willaert 2021a). Alternatively, and more likely, US-owned subsidiaries incorporated in Member States will provide a ‘back door’ through which the USA will be able to actively participate in DSM (DSM Observer 2020a). Lockheed Martin, a US-incorporated company, indirectly holds exploration contracts through its UK-registered wholly owned subsidiary UK Seabed Resources, due to the USA being unable to sponsor its own firms (Casson et al. 2020). Despite other possibilities, the ambiguity over the exact legal status of US-based firms engaging in DSM activities to exploit PMNs is in itself potentially highly problematic for the alleviation of cobalt supply security for US markets, and will likely remain so until the USA joins the ISA by ratifying UNCLOS.

### Political barriers to DSM of PMNs

The mineral resources of the Area are designated to be “the common heritage of all mankind”, according to Article 136 of UNCLOS, mandating that their exploitation should “be carried out for the benefit of mankind as a whole, irrespective of the geographical location of States”. In a Westphalian system of global organisation, where states act first in self-interest, and second in the interest of ‘mankind as a whole’, competing objectives between and amongst states will inevitably result in political disharmony (Hickey 2020). Despite enduring political stability (Kirkpatrick 2007), the United Nations has a poor record on major cross-border issues such as climate change (Napoli 2012), the COVID-19 pandemic (Guterres 2020), and indeed enforcement of provisions within UNCLOS itself (Hayton 2014; Afrin 2017). The lack of unanimously observed authority encourages powerful states to ignore or even outright reject decisions made by UN bodies relating to matters over which they have total jurisdiction^6^ (Kardon 2018). In practice, this introduces new dimension of risk factors associated with geopolitics to DSM in the Area, ought to alert operators to the political realities of working within the Area.

Unlike terrestrial mining activities, DSM operations in the Area cannot be undertaken by a private corporation alone, with a requirement that any contractors exploiting mineral resources be sponsored by a State Party to UNCLOS (Lily 2018). This ties contractors to a State, and whilst this may provide them with financial and physical security, contractors must be wary that there may be expectations from that State in exchange. This potential for political interference from sponsor State may take many forms, with states often having a wider range of objectives than the commercial entity’s fundamental bottom line (Lan and Rainey 1992, and references therein). For operators with ultimate US ownership requiring the sponsorship of a different State, this probability of political interference may be greater than for others sponsored by their State of incorporation; if a country has no domestic mining industry to protect, it may display less concern for the health of a foreign owned entity, and thus be more willing to threaten to terminate sponsorship if interference is resisted by the contractor. There are no provisions in either the Prospecting and Exploration Regulations (International Seabed Authority 2013) nor the Draft Exploitation Regulations (ISA 2018) requiring reasonable justification for termination, essentially allowing the sponsor State to terminate sponsorship at its convenience. Though the contractor may be able to financially mitigate against this eventuality with political risk insurance (Webb 2012), it is nonetheless a risk of which they must be cognisant.

**Fig. 6:**
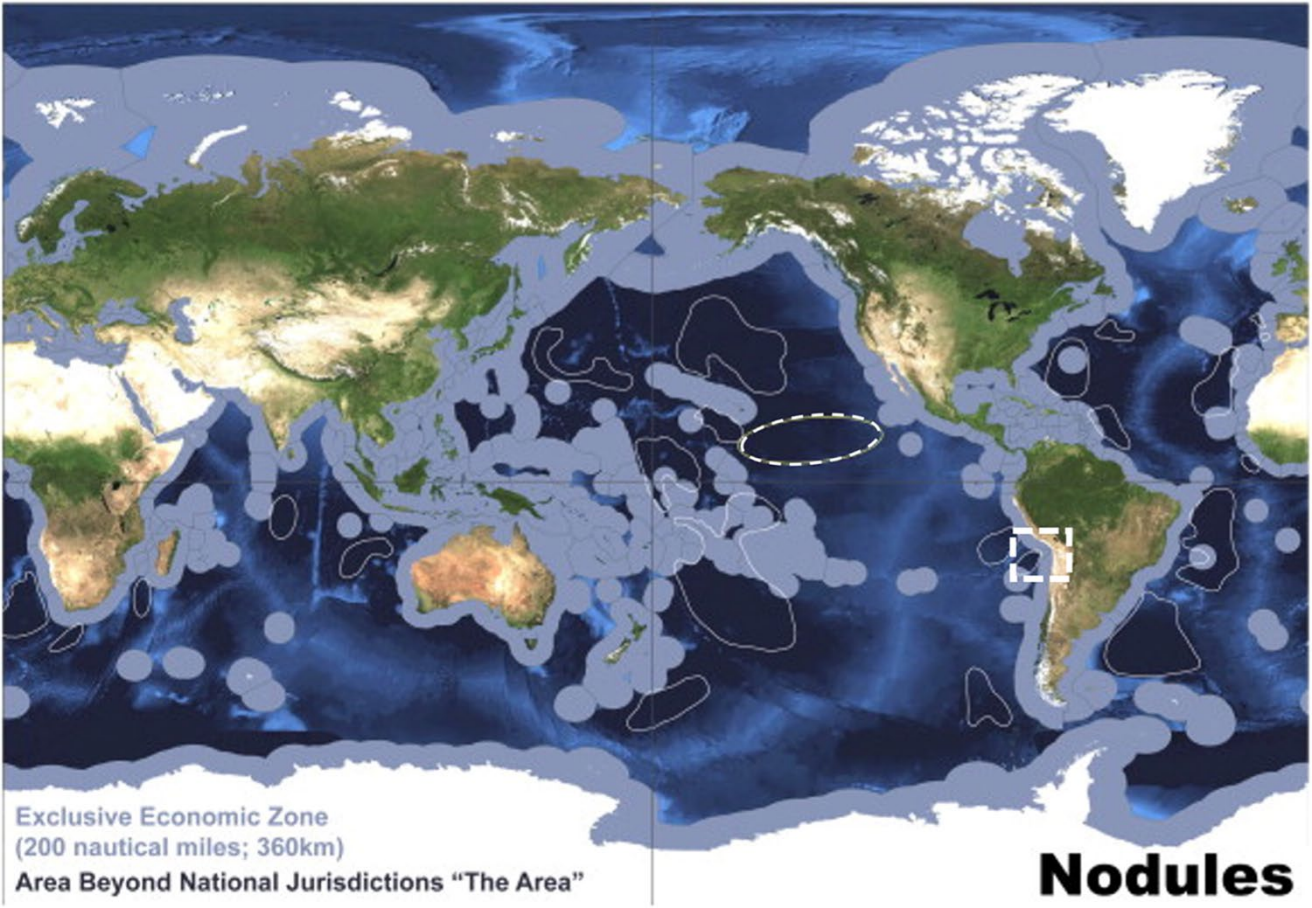
Map of global distribution of EEZs (blue-grey shading around land), competing territorial claims (dash-bordered square (not to scale)), and global permissive areas for development of abyssal plane PMNs; the CCZ is the area of greatest economic interest (dash-bordered oval (not to scale)); all other areas are marked with a solid white border. A permissive area does not guarantee the presence of economic PMN deposits. Small, dispersed PMN deposits will occur elsewhere. Adapted from Hein et al. (2013) with permission. © Elsevier

**Fig. 7:**
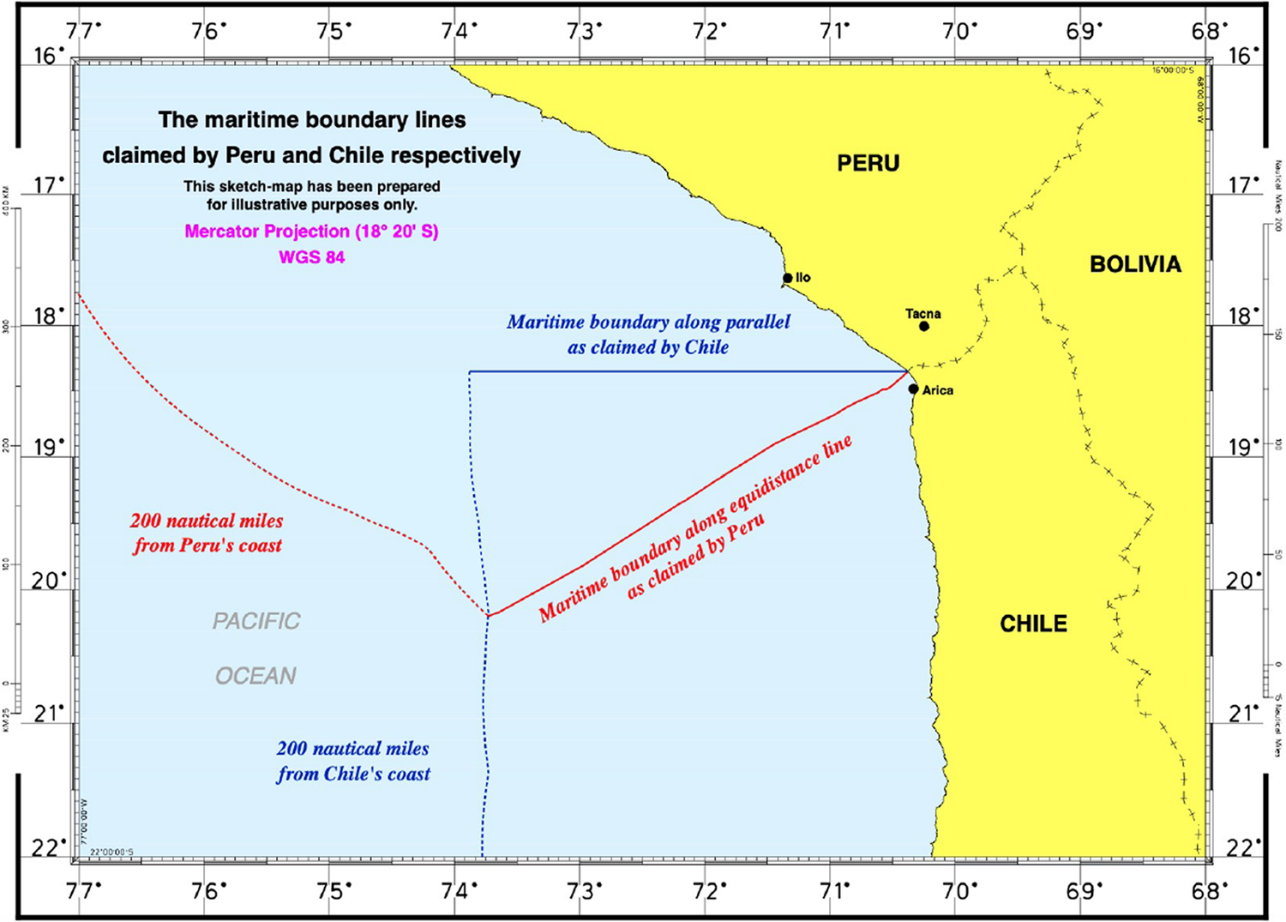
Map displaying competing maritime territorial claims by Peru and Chile, area denoted in Fig. 6 by white dashed square (area in Fig. 6 is not to scale, and is drawn to indicate general region). After International Court of Justice (2014)

Political risk is not limited to state interference threatening operational independence of the contractors. When a contractor is sponsored by a State, it is seen, to an extent, to represent that State. Though this can provide protection if the sponsoring State is sufficiently feared and/or respected, there is also the possibility that the operators and their equipment may be seen as a legitimate target for aggression by hostile actors aiming to harm the sponsoring State. In 2011, an exploration ship operated by a British-based petroleum exploration company was targeted by Chinese vessels for surveying in waters considered to lie in the Filipino EEZ by UNCLOS, though claimed by PRC (Hayton 2014). This incident, which arose due to the nationality of the issuer of the exploration licence, resulted in the company considering abandoning operations in the area altogether (Fabi and Mogato 2012). At present, there are few instances of overlap between areas for PMN development and areas subject to rival maritime claims by sovereign states (Fig. 6; Fig. 7), reducing the potential for similar conflicts in relation to PMN deposit development. This is no guarantee, however, that conflicts will not arise in the future, and thus contractors ought to be wary of inadvertent involvement in territorial disputes when evaluating potential DSM activities.

## The case for deep-seabed mining of PMN deposits

Deep-seabed mining has been discussed with enthusiasm since geologist John Mero attempted to calculate the economic value of PMN deposits in the oceans, publishing his findings in The Mineral Resources of the Sea (Mero 1965; Meyer et al. 2019). An initial swell of excitement was dampened with a fall in commodity prices and the proscription of DSM by UNCLOS in 1982 (Sparenberg 2019; Chin and Hari 2020). Despite these early setbacks, advances in technology, combined with a surge in demand for critical minerals such as cobalt, have turned attention back to the deep-seabed and PMNs in particular as an alternative source for cobalt, manganese, copper, and nickel (Childs 2020). Recent projections over the past decade and a half have reiterated the imminence of DSM (Halfar and Fujita 2007; Allsopp et al. 2013; Heffernan 2019), with Nauru’s triggering of the ‘two-year rule’ possibly commencing the overcoming of the largest regulatory barrier to date (Thaler 2021). The possibility of commercial DSM activity has evolved through the form of a distant possibility to a near inevitability (Carrington 2017). The reasons for this are explored in this section. Whilst this paper aims to maintain objectivity, it is vital to explore the reasons why DSM for PMNs is in the process of becoming an unavoidable reality.

### The environmental argument

It is widely accepted that the demand for cobalt is increasing in response to growing environmental concerns, mainly due to its role in the energy transition (Alves Dias et al. 2018; Chen et al. 2019). This places global societies and economies in a difficult predicament between continuing to use hydrocarbons, and trying to meet escalating demand for a material with already limited supply. The environmental forecasts of the current situation are grave, with supply-side issues rendering cobalt an unstable foundation on which to build the transition to a low-carbon economy. Current hydrocarbon and cobalt extractive operations and supply chains are far from environmentally harmless (Ahmad et al. 2017; Otamonga and Poté 2020; van den Brink et al. 2020), rendering the global choice not between hydrocarbons and cobalt for the energy transition, but between the present trajectory of inadequate environmental action and a potential solution. It must be acknowledged that society has just as much agency in the decision to maintain the status quo as it does to change — inaction is as conscious a choice as action. This is no simple choice, and any solution will inevitably involve some environmental harm, as discussed in the “Environmental barriers to DSM of PMNs” section.

Though environmental comparisons ought to be made with caution due to technical, situational, and ecological differences, there are undoubtedly upsides to DSM for PMNs, when compared with terrestrial cobalt mining. Notably, PMNs are composed of almost 100% theoretically economically marketable materials^7^ (Sommerfeld et al. 2018), in comparison to terrestrial ores, which often yield less than 1% of marketable materials (Toro et al. 2020a). This has the potential to reduce solid waste in the form of tailings, a major source of mine pollution (Licskó et al. 1999; Holland 2020), by two orders of magnitude from the terrestrial equivalent (Toro et al. 2020a). Despite difficulties in comparisons, it is believed that “DSM could pose significantly less environmental harm than land-based mining”, whilst bringing the same benefit by way of the mineral in question (Bolong 2016).

Whilst accepted to be an anthropocentrically orientated argument, there is also the opinion that the more intense the environmental effect on human existence, the worse the outcome. Expanding on this viewpoint, the severity of the environmental effects of terrestrial cobalt mining on local communities arguably reinforces the case to reduce this by transitioning to much less immediately anthropologically detrimental PMN-sourced cobalt (Nogrady 2020). At present, the detriment to local environments of cobalt mining, and the wider climate of hydrocarbon extraction and usage foment urgency to transition to an alternative, presented in the form of DSM. Society must seriously consider the risks to the marine environment of DSM activities prior to its introduction (the “Environmental barriers to DSM of PMNs” section), but must also deliberate the implications of not pursuing PMN extraction. The effects of GHG-induced warming on the wider climate and consequently, marine environments have been well documented (McMichael et al. 2004; Brierley and Kingsford 2009). This leaves the decision between allowing marine environments to suffer the effects of climate change globally, or sacrificing the stability of some local marine environments through DSM for the health of marine ecosystems worldwide, as well as the wider climate.

In a broader context, a greater supply of cobalt to the world economy would theoretically reduce its price on global markets, or at the very least, stabilise it in the longer term. This would, in turn, provide more solid foundations for the energy transition, as well as reducing the unit cost of lithium-ion batteries (Alves Dias et al. 2018), which would arguably give a considerably higher net environmental benefit than if DSM were not to take place. Seen through the lens of criticality, it is vital that supply concerns for cobalt are eased in the USA, which is the second largest manufacturer of EVs behind PRC (Carlier 2021). With cobalt supply previously, and potentially again, a key bottleneck in the production of EVs (Kobie 2020), it is arguable that the health of the environment, to an extent, relies on supply security of cobalt to US markets. Some may suggest that this shifts the debate regarding the necessity for and acceptance of DSM, to a question of how long the environment and wider climate can survive without the addition of PMN deposits to the cobalt reserve base.

### The political argument

In 1973, the USA experienced an energy crisis rooted partially in its reliance on imported oil from nations with which there were disagreements on foreign policy. Though the exact causes are still disputed, the importance of raw material supply security was thrust into the spotlight and became a national concern (Lifset 2014). There may be fundamental differences between crude oil and cobalt, and whilst a cobalt shortage would not directly affect consumers in the same way the energy crisis did, cobalt supply must still be a vital political consideration for US policymakers. More pertinently, the recent conflict in Ukraine has caused supply shocks to US markets for both manganese and nickel (both constituent metals in PMNs), with Ukraine accounting for roughly 10% of the world’s manganese reserves (Schnebele 2022) and Russia producing over 10% of the world’s nickel in 2020 (McRae 2022). Supply diversification of nickel and manganese as well as cobalt away from less politically stable regions, through the introduction of DSM, strengthens the political imperative to the USA of exploiting PMN deposits.

Given the growing importance of cobalt to the US economy, as well as its increasing criticality (Beatty et al. 2019), the USA must, and likely will, be proactive and decisive, in order to pursue intelligent long-term strategies and manage world politics to improve their position (Cable 1995; Kane 2006). In essence, the ideal for US supply security would be that it controlled the entire supply chain from extraction to manufacture. Failing this, it ought to seek to negate control from states who may yield it as a coercive tool against the USA. When the USGS published America’s first formal critical minerals strategy in 2019 (Executive Order 13,817), then Interior Secretary David Bernhardt stated that they were “dedicated to ensuring that we are never held hostage to foreign powers for the natural resources critical to our national security and economic growth” (USGS 2019). The current market structure for cobalt is not favourable to the USA, leading to its classification as a critical raw material (“Introduction and global significance” section). Reliance on foreign states and enterprises for the cobalt supply reduces US political influence over its own economy, and it must therefore explore avenues to improve the situation.

Over recent decades, political tensions between the USA and PRC have grown substantially (Brattberg 2021). At the same time, PRC has sought to dominate cobalt’s supply chains, securing its own access to cobalt at the expense of US markets (Matthews 2020). Other than improving recycling, which will be insufficient alone to secure supply to required levels (Church and Wuennenberg 2019), a political solution in the form of active DSM endorsement is arguably now necessary. PRC’s policy of a concerted effort to secure cobalt supply for domestic markets demonstrated the effectiveness of co-ordinated government intervention. In comparison, the previously laissez-faire attitude to cobalt access from US policymakers has resulted in it being left behind, seeking an alternative source of cobalt in order to stave off supply concerns (Savage 2020). Bolstering the political imperative to introduce PMN exploitation for cobalt supply are the parallels with Fig. 6, also contained in PMNs, with China responsible for around 60% of total REE production in 2020 (Cordier 2022). REEs are considered extremely important to the US economy for use in a wide array of applications including production in high-tech manufacturing (Cohen and Grant 2021), and were recently included alongside cobalt in a press release relating to the Biden administration’s investment to “expand domestic critical minerals supply chains, breaking dependence on China” (The White House 2022).

DSM provides the USA with an invaluable opportunity to reverse the deficit of influence in the cobalt market and secure its own supply chains. Though there are difficulties regarding its membership of the ISA, as outlined in the “United States specific barriers” section, US firms are still in a favourable position to secure cobalt from PMN resources for domestic markets. If companies based in the USA or allied states are able to access these resources, the criticality of cobalt will be significantly decreased. This control must not be limited to DSM operations alone, however, and active policies must be enacted to encourage refining and processing in the USA. This would then allow the USA greater freedom to pursue foreign policy goals which may conflict with those of PRC, and would previously have encouraged PRC to threaten cobalt supply to the USA.

This being said, the USA must be wary of appearing to be attempting to circumvent UNCLOS provisions. Though it has not ratified UNCLOS, it iterates its expectation that PRC adheres to UNCLOS rules (United States Secretary of State [Michael R. Pompeo] 2020), justifying this expectation under the principle of customary international law (Duff 2005). If it is seen that the USA are not abiding by UNCLOS rules, regardless of their applicability to US activities, this may encourage PRC to escalate its claims over territory, and thus the contained resources, within the South China Sea and beyond (Colin 2016).

### The economic argument

The entire rationale for DSM of PMNs in the Area as a method of alleviating supply-side issues for critical minerals to US markets, such as cobalt, is economic. On the most rudimentary level, there is a supply-side bottleneck which can be rectified by an alternate source, facilitating a more efficient allocation of resources. Of course, the realities are much more complex, even when all factors other than the economic aspects are discarded.

**Fig. 8:**
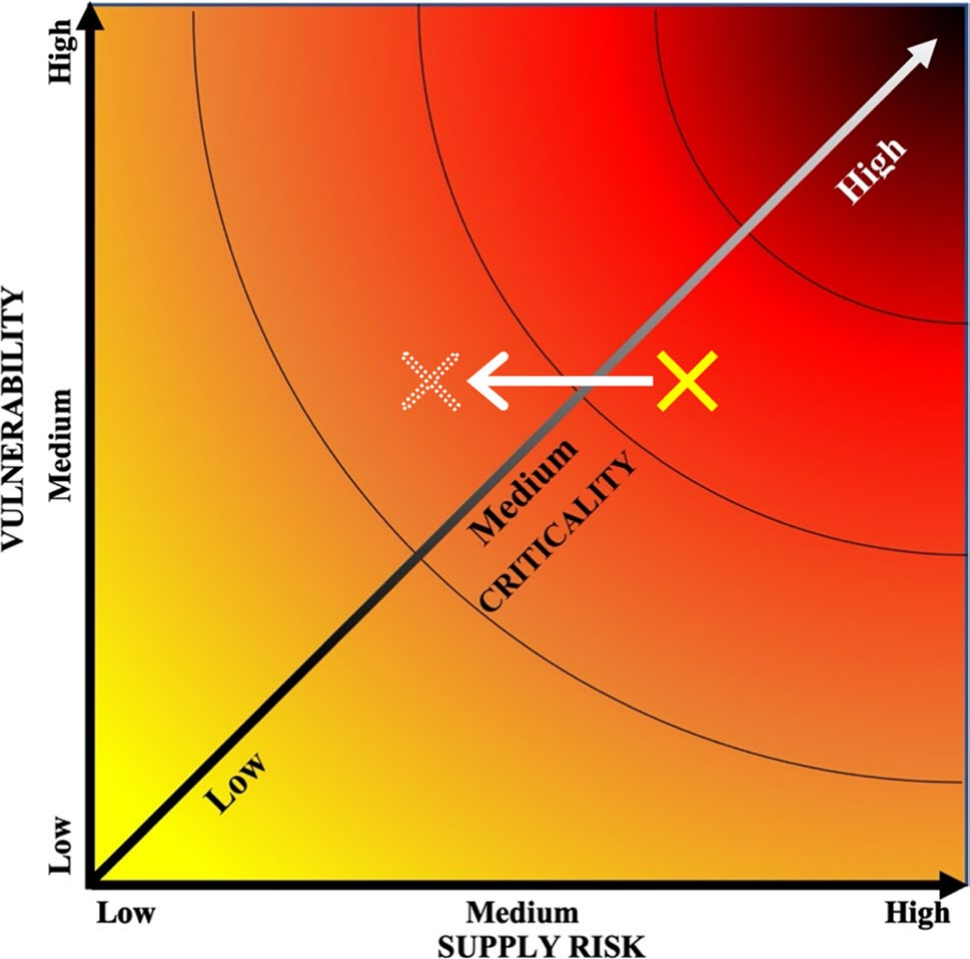
Illustrative criticality matrix showing relative decrease in criticality of cobalt due to drop in supply risk resulting from the introduction of cobalt recovery from PMN sources. Current cobalt criticality is denoted by solid cross. Expected cobalt criticality with DSM of PMNs is denoted by dotted-outline cross. After National Research Council (2008)

The backbone of the economic argument for cobalt recovery from PMNs is rooted in the economic concept of criticality, denoting demand inelasticity (vulnerability/importance to an economy) and capability of the market to ensure supply (supply risk). Despite attempts to decrease reliance on cobalt for battery technology, cobalt-bearing batteries are expected to continue to dominate the EV battery market (Ryu et al. 2021), maintaining demand inelasticity. Though DSM for PMNs will not reduce the importance of cobalt to the US economy, by strengthening supply security, it can undoubtedly reduce overall criticality, as illustrated in Fig. 8.

The economic viability of DSM for PMNs remains unproven in practice, though this is at present due more to a prohibitive regulatory framework than pervading insurmountable technical or wider economic challenges. Multiple recent studies have suggested PMN mining in the Area is economically feasible in theory (Volkmann and Osterholt 2017; Roth et al. 2018b; Mukhopadhyay et al. 2019), although calculations are made on the assumption of a single operator working with current market conditions. Roth et al. (2018b) note that one seabed operator may have visible effects on global copper and nickel prices, which may mean that multiple operators greatly increasing global cobalt supply could significantly reduce cobalt price to a theoretically subeconomic level. As cobalt is seen as an economic by-product (Mukhopadhyay et al. 2019) in PMNs, a drop in price, as a result of supply increase from PMN reserves, could end up filtering through the US economy. Given the relative similarity and uniformity of PMN deposits (Hein et al. 2013), it is likely that proven economic feasibility of DSM for PMNs will allow for the development of an extensive share of PMN deposits. This will likely increase supply by a large amount and result in a correspondingly large decrease in price. This would make components and consumer products containing cobalt cheaper, potentially improving the performance and long-term security of US manufacturing industries, as more operations become economically viable.

## Conclusions

Deep-seabed mining may be the biggest development in the extractive sector of this century, with strong discussions over its political, economic, and environmental necessity and viability. Reminiscent of the environment in which it is expected to take place, there is much more to know than that which is already known. When assessed as a method of alleviating supply-side issues for cobalt in terms of feasibility, it appears to stand up to scrutiny, though is far from a panacea. With the growing reliance on lithium-ion batteries, of which cobalt is and will likely remain a key component, for use in the energy transition, the question of cobalt’s correspondingly increasing criticality is becoming difficult to ignore.

The Area as a jurisdiction and accompanying regulatory framework offer some respite to the tumultuous situation surrounding terrestrial cobalt mining. Currently, 68% of the world’s cobalt is produced in DRC, where rule of law is weak, institutional strength low, and political stability turbulent with cobalt. Refining is also heavily concentrated in PRC, which brings its own unique challenges in relation to governance and political risk, sitting in 100th place overall of 140 countries on the World Governance Indicator Index (World Bank 2021) (DRC is ranked 139/140). There are a multitude of reasons why the criticality of cobalt would be greatly decreased by the introduction of cobalt from PMN deposits into the supply chain. As noted in the “The political argument” and “The economic argument” sections on the political and economic arguments for PMN extraction, respectively, these come about not only due to supply diversification in more general terms, but as a greatly contrasting alternative to the current precarious supply chain dominated by two jurisdictions which are each considered problematic for their own unique reasons.

Though there are reasonable concerns over the enforcement capabilities of an intergovernmental body such as the ISA, which derives its legitimacy from the UN, it is comparatively seen as a much more politically stable jurisdiction than DRC. However, there are currently no published exploitation regulations for PMN mining in the Area, leading to concerns as to the industry’s prospects of success. This being said, a degree of regulatory uncertainty will likely dissipate somewhat upon publication of the Regulations, expected by June 2023, after Nauru’s triggering of the two-year rule in June 2021.

Economic debates surrounding DSM are numerous and expansive, ranging from the technical feasibility of economic extraction to the viability of relying on the long-term economic health of multiple metals. The novelty of the field of PMN mining brings considerable economic uncertainties, resulting in expansive predictions reliant on precarious assumptions. It is expected that substantial capital and operational expenditure will be required, contributing to its characterisation as a high-risk, high-reward endeavour, when compared with terrestrial mining operations. The lack of formal exploitation regulations has undoubtedly stymied investment so far, and whilst there are significant theoretical upsides, the markets have so far deemed the risk-reward profile unpalatable.

These economic questions feed into political issues surrounding DSM of PMNs as a solution to cobalt criticality in relation to the USA, with criticality, as a concept, being an amalgamation of economic and political considerations. Though economic vulnerability and supply issues are the problem, it is arguable that the USA is in dire need of a political solution. American firms are not ostensibly permitted to engage in DSM, with the US membership, or lack thereof, of the ISA prohibiting it from acting as a Sponsor State to DSM contractors. There are, of course, workarounds, including foreign-registered subsidiaries and strategic partnerships with allied states, although these solutions each bring political challenges of their own. The growing US rivalry with PRC will lead to further scrutiny of its behaviour, though also heightens the need to decrease US economic reliance on PRC. This includes cobalt supply, for which the USA must find alternatives to PRC-dominated supply chains, in order to avoid being “held hostage to foreign powers for the natural resources critical to [their] national security and economic growth”.

Though any substitute to the current supply chain is welcome from a criticality perspective, there are wider considerations that must be taken into account regarding the viability of DSM of PMN deposits as an alternative. There are a plethora of questions which must be answered prior to the initiation of DSM in the Area that encompass a variety of subjects from the environmental impact to the economic and regulatory feasibility. The main opposition to DSM activities regards its potential environmental impacts (the “Environmental barriers to DSM of PMNs” section). As yet, there is simply not enough information to discern the range and severity of detrimental effects that DSM for PMNs will have on the deep-sea environment. The stillness and sensitivity to change of the abyssopelagic environment is likely to amplify any physical, biological, and chemical changes to the aquatic surroundings. The exact changes that will be made to this environment by DSM for PMNs, as well as the biological response to these changes, is alarmingly poorly understood, leading to many suggestions that a moratorium ought to be placed on DSM activities until more is known. Arguments against this, outlined in the “The environmental argument” section, are based in pragmatism, and suggest that the wider environment is suffering as a result of inaction. Insufficient adoption of electric vehicles due to high cobalt prices as well as the environmental issues associated with terrestrial mining are, as advocates of DSM suggest, considerably worse than the local environmental impacts of PMN extraction.

When deep-seabed mining of polymetallic nodules in the Area is assessed as a method of alleviating supply-side issues for cobalt, through the perspective of its status as a critical mineral, the pragmatic choice suggests that it is a viable and appropriate course of action. It must not, however, be relied on alone to correct the issues that contribute to cobalt’s criticality to the US economy. Rather, it should be used as the foundation of a wider policy to ensure that the cobalt supply chain is less dominated by external powers, especially adversaries such as PRC. Cobalt’s value is partially derived from its indispensability to the energy transition, important in rectifying global environmental damage from fossil fuels. The adverse environmental impacts of DSM for PMN-hosted cobalt must therefore be closely monitored in order to avoid negating the environmental benefits it brings. There is still no determined answer on whether the urgent requirement for cobalt’s benefits justifies the introduction of PMN mining before there is sufficient knowledge on its environmental impacts. There is, however, clear impetus for its immediate introduction from an economic and political standpoint, in order to alleviate issues around cobalt supply security to US markets. Whilst DSM for PMN deposits in the Area appears to be inevitable, its exact technical and economic viability remains to be seen, and its overall efficacy as a strategy for improving supply security may not be determined until the ISA allows commercial operators to take the plunge.

## Footnotes

1 Reserve base includes those demonstrated (measured plus indicated) resources that are currently economic (reserves), marginally economic, and subeconomic (USGS 2022).

2 The Solwara 1 project aimed to mine seabed sulphide deposits from hydrothermal vents in the territorial waters of Papua New Guinea, which is technically a very different operation to PMN extraction. Despite the differences between this and PMN extraction in the Area, it may still be seen by investors as too closely associated with DSM of PMNs.

3 The metallurgical plant is expected to comprise the majority of the necessary CAPEX and OPEX due to its size, specificity, and running costs.

4 Although contractors may originate from one country (Country A), and be sponsored by a second country (Country B), their support and/or extraction vessels may be registered by a third country (Country C), which is responsible for ensuring the compliance with provisions of UNCLOS, according to Article 217 (1) (UN 1982).

5 Article 161 of UNCLOS mandates 36 members, although there are currently 37 members for regional equity.

6 PRC refused to participate in arbitration sought by the Philippines under UNCLOS’ compulsory dispute resolution procedures in 2013 relating to PRC claiming sovereignty over areas of the South China Sea considered to lie within the Philippines’ EEZ by UNCLOS (Pemmaraju 2016).

7 This figure assumes a four-metal extraction method, which recovers manganese alongside copper, nickel, and cobalt. There is also the assumption that the market has sufficient demand for ferromanganese, a by-product of the extraction process documented by Sommerfeld et al. (2018).

## Declarations

### Confirmations

I confirm that all authors have approved the manuscript for submission.

I confirm that the content of the manuscript has not been published or submitted for publication elsewhere.
